# Quantifying the impact of simple DNA parameters on the cyclization *J*-factor for single-basepair-addition families

**DOI:** 10.1038/s41598-018-22502-7

**Published:** 2018-03-20

**Authors:** Yunjin Tong, Robert S. Manning

**Affiliations:** 1 The Shipley School, Bryn Mawr, PA 19010 USA; 2 Haverford College, Department of Mathematics and Statistics, Haverford, PA 19041 USA

## Abstract

We use Monte Carlo simulation to quantify the change in cyclization *J*-factor within a dramatically simplified model of DNA that involves parameters for uniform stiffnesses, intrinsic twist, and intrinsic bending (including nonplanar bending). Plots of *J* versus DNA length over multiple periods of helical repeat are fit to a simple functional form in order to project the behavior of *J* over a broad range of these model parameters. In some instances, this process allows us to find families of DNA molecules (within our model) with quite different material properties, but very similar plots of *J* versus length, so similar as to likely to be indistinguishable by experiments. This effect is seen both for the parameter-pair of bend angle and stiffness scaling, as well as for the parameter-trio of helical repeat, bend angle, and bend non-planarity.

## Introduction

The propensity of a short DNA molecule to cyclize, often measured experimentally via the *J* factor, has long been used as a probe for basic DNA structural properties like stiffness and curvature^1–4^. Many experimental designs have involved measuring *J* for a family of DNA that share a common sequence but add a small number of basepairs^5–7^.

In this article, we perform Monte Carlo computations that correspond to this style of experiment, computing what we will call the *cyclization profile* of a DNA sequence: the plot of *J* versus DNA length when adding single basepairs to the sequence under study. In each case, we add 1–25 basepairs, so that the profile contains a bit more than two full periods of the oscillation due to the intrinsic twist of the DNA, enough to fit a smooth curve through the results and extract the most prominent features, especially the heights and locations of the peak and troughs.

Our computational goal is to obtain highly accurate estimates of *J*-factors (within 0.1 in log_1__0_ *J*) for each cyclization profile as we vary several shape and flexibility parameters in the DNA. From these computations, we find several degeneracies in the problem, in the sense that DNA with quite different shape/flexibility parameters produce cyclization profiles that are nearly indistinguishable. These degeneracies pose a substantial challenge to the basic inverse problem of taking cyclization experimental results and extracting from them conclusions about the DNA’s mechanical properties.

In terms of the dependencies of the *J*-factor on each parameter studied, many of the effects are intuitive, but still its quantification allows a useful “calibration”, e.g., one can take *J*-factor computations coming from recent models with hundreds or thousands of parameters and align them with a simple model with just a few, more intuitive, parameters. We demonstrate one such calibration below. In addition, the dependence of the *J*-factor on a few of our parameters is less intuitive, and we highlight those results as potentially interesting for further study.

The computational exploration of the dependence of *J* on DNA length is hardly unique to this article. In addition to the works cited above, many of which paired experimental results with computational studies akin to our approach, there is also the early work of Shimada and Yamakawa^8^, who studied the dependence of *J* over a long range of lengths for uniform unbent DNA, and Levene and Crothers^9^, who translated the statistical-mechanical theory of *J*-factors from Jacobson, Stockmayer, and Flory *et al*.^10,11^ into a Monte Carlo computation that is related to our approach. More recently, there was the study by Zhang and Crothers^12^, which included results relating *J* to curvature and helical repeat, with *J* computed via a new harmonic approximation, the work by Towles *et al*.^13^, which used Monte Carlo computations to study *J* as a function of length for looping in the presence of Lac repressor, along with other studies of this type. Our study is particularly connected to Czapla, Swigon and Olson^14^, both in methodology (as we use an adaptation of their version of the Alexandrowicz^15^ half-molecule trick to develop high-accuracy computations) and in a focus on the dependence of the cyclization profile on a few relatively simple parameters (although our parameters of study differ).

We aimed to make our results distinctive from this literature to which we are indebted by focusing on quantifying the dependence of the cyclization profile on each of our parameters (over a fairly wide range of values relevant to DNA). This quantification can allow our simplistic-model results to serve as a potentially useful calibration for results coming from a more intricate model. Furthermore, through that quantification, we are also able to demonstrate what we believe is an important underlying degeneracy in the cyclization *J*-factor, namely that one can get very similar results (so similar as to be beyond the current ability of experiments to distinguish) from DNA molecules with very different material properties.

## Results

In most of the results that follow, cyclization profiles are analyzed by fitting to the piecewise-quadratic functional form seen in Eq. (29). For convenience, we summarize in Table 1 here the meanings of the five parameters that appear in that functional form:

**Table 1:** Parameters that appear in the functional form used to fit cyclization profiles.

Parameter	Meaning
*ϕ*	Phase-shift (specifically, basepair location of first peak after *N* = 145)
*p*	Period of oscillation (in basepairs)
*a*	Constant vertical shift (specifically, value of log_10_ *J* at first peak)
*b*	Linear drift (specifically, rise in log_10_ *J* from peak to next peak)
*c*	Quadratic coefficient (controls peak-to-trough difference)

### Intrinsic bend and stiffness are likely to be difficult to distinguish experimentally

When computing cyclization profiles coming from a single bend, or from a shift in stiffness, we see similar effects. To make this concrete, we use our piecewise-quadratic functional form (see Eq. (29) in Methods) to generate a family of molecules with different mixes of intrinsic bend and stiffness but nearly identical cyclization profiles.

#### Effect of planar intrinsic bend

**Figure 1:**
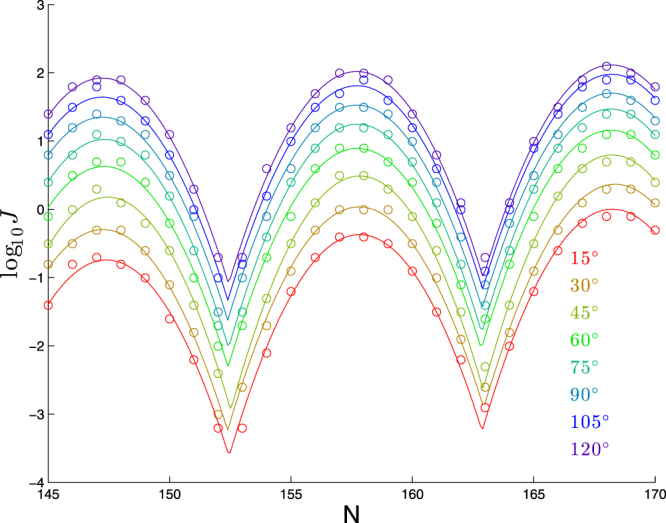
Dependence of cyclization profile on bend angle. Each molecule consists of a planar bend (varying from 15° to 120°) over 63 bp followed by a straight segment of (*N* − 63) bp.

We show in Fig. 1 the effect of varying the bend angle within a molecule consisting of a 63-bp planar bend followed by a straight segment. (See the Supplementary Material for results demonstrating that shifting the bend to start later in the molecule has no signficant impact on the results).

By eye, the main effect is a constant shift in log_10_ *J* with increasing bend angle. This is confirmed by fits to our functional form (29), as seen in Table 2, where we see a clear upward trend in the vertical shift parameter *a* from (29) with bend angle.

**Table 2:** Best-fit parameters for each curve in Fig. 1 to log_10_ *J* = *a* + *b*(*N* − *ϕ*) + *c*[*f*(*N*, *ϕ*, *p*)]^2^.

Bend angle (in°)	*ϕ*	*p*	*a*	*b*	*c*
15	147.24	10.42	−0.74	0.036	−0.113
30	147.12	10.55	−0.30	0.031	−0.112
45	147.33	10.39	0.18	0.030	−0.121
60	147.20	10.43	0.63	0.025	−0.113
75	147.22	10.43	1.02	0.021	−0.117
90	147.16	10.48	1.35	0.017	−0.112
105	147.16	10.49	1.64	0.016	−0.111
120	147.19	10.48	1.92	0.009	−0.112

Table 2 also shows a downward trend in the linear-drift parameter *b* from (29). However, since the maximum value of *N* − *ϕ* is 23, the absolute impact of the change in *b* is less than 0.027 × 23 ≈ 0.6 in log_10_ *J*, as compared to the overall shift by 2.6 in log_10_ *J* due to the change in *a*. The other three parameters *ϕ*, *p*, and *c* that appear in (29) do not appear to show any significant trends.

**Figure 2:**
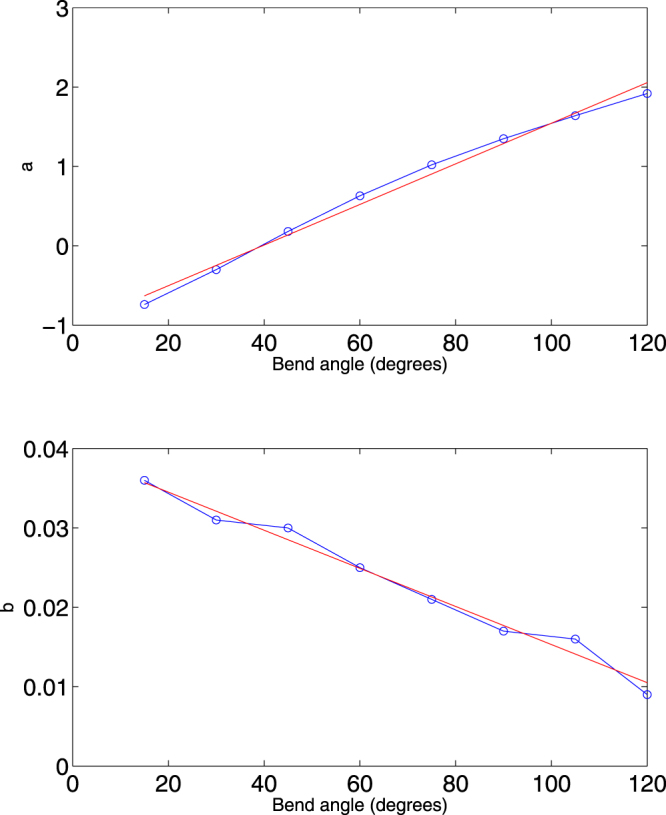
Dependence of the coefficients *a* and *b* on bend angle (blue circles) and linear regression fit (red line) from Eq. (1).

To quantify the effect of bend angle on the cyclization profile, we apply linear regression to the values of *a* and *b* from Table 2, finding:

1 $$a\approx -1.02+0.026({\rm{Bend}}\,{\rm{angle}}),\quad b\approx 0.040-0.0002({\rm{Bend}}\,{\rm{angle}});$$

see Fig. 2 for plots corresponding to the regression Eq. (1). We see from the plot of *a* versus bend angle a systematic secondary effect in the downward curvature of the plot. To quantify this effect, as compared to the overall best-fit slope of 0.026 seen in Eq. (1), the best-fit slope in the range 15° ≤ Bendangle ≤ 60° is 0.031, whereas the best-fit slope in the range 75° ≤ Bendangle ≤ 120° is 0.020.

Summarizing, the primary effect of increasing bend angle is for the cyclization profile to shift upwards by 0.1 in log_10_ *J* for each 4 degrees of bend (within our construct of a 63-bp planar bend). We also have some evidence of secondary effects: the decrease in *b* with bend-angle and downward curvature in the plot of *a* versus bend angle.

Since our goal is to focus in general terms on how key physical features of the DNA impact the cyclization profile, we will minimize our consideration of secondary effects. To this end, for the remainder of this paper, we will restrict our attention to the regime 60° ≤ Bendangle ≤ 120°, which also has the benefit of computational efficiency, since larger *J*-factors can be computed more quickly. Applying linear regression to *a* for just this range yields the fit:

2 $$a\approx 1.31+0.021({\rm{Bend}}\,{\rm{angle}}-90)\mathrm{.}$$

Our study of a wider range of bend angles in this section suggests that the results we will find for 60° ≤ Bendangle ≤ 120° would carry over with only minor changes to cases where Bendangle < 60°.

#### Effect of overall shift in stiffnesses

**Figure 3:**
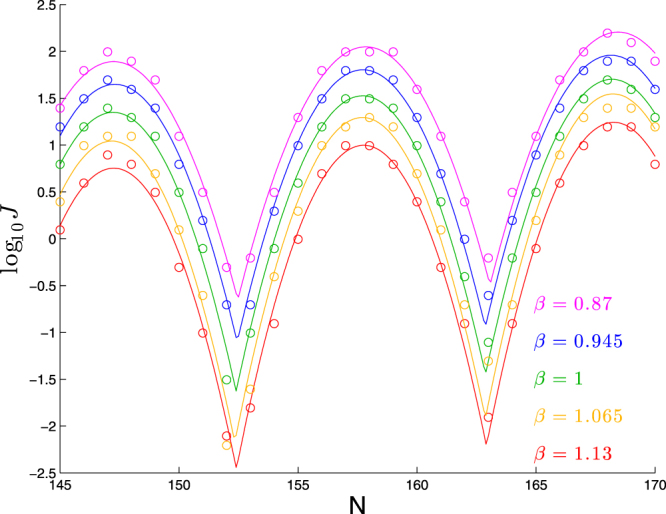
Dependence of cyclization profile on a factor *β* that scales all six stiffnesses. Each molecule consists of a planar bend of 90° over 63 bp followed by a straight segment of *N* − 63 bp. All six stiffnesses shifted by the same factor.

We show in Fig. 3 the effect of scaling all six stiffnesses by a factor *β*. For the purposes of calibration, we note that the values of *β* used would correspond to persistence lengths of 40.3, 43.3, 46.3, 49.3, and 52.3 nm if the DNA were intrinsically straight.

As we saw when changing bend angle, the main effect of scaling the stiffnesses is a constant shift in log_10_ *J*. This is confirmed by fits to our functional form shown in Table 3, as now the parameters *ϕ* and *p* show no consistent trend, the parameters *b* and *c* show mild trends (one upward, one downward), and the parameter *a* shows a clear downward trend.

**Table 3:** Best-fit parameters for each curve in Fig. 3 to log_10_ *J* = *a* + *b*(*N* − *ϕ*) + *c*[*f*(*N*, *ϕ*, *p*)]^2^.

*β*	*ϕ*	*p*	*a*	*b*	*c*
0.87	147.17	10.59	1.89	0.015	−0.093
0.945	147.23	10.43	1.65	0.015	−0.104
1	147.16	10.48	1.35	0.017	−0.112
1.065	147.08	10.53	1.04	0.024	−0.121
1.13	147.16	10.50	0.75	0.023	−0.121

As we saw with bend angle, the *impact* of the variation in *b* is minimal: its variation by 0.008 would only make for a change in log_10_ *J* of 0.008 × 23 ≈ 0.2. On the other hand, the variation in *c* has a larger impact: the change in *c* by 0.03 will have no impact on the peaks of the cyclization profile, but will shift the height of the troughs by 0.03 × (5.5)^2^ ≈ 0.9 (hence the purple curve in Fig. 3 has trough-to-peak variation of about 1.8 − (−0.6) = 2.4 whereas the red curve has trough-to-peak variation of about 0.7 − (−2.5) = 3.2).

Applying linear regression to the best-fit values of *a*, we find:

3 $$a\approx 1.34-4.45(\beta -1).$$

Thus, in this range of *β*, the primary effect of increasing *β* is for the cyclization profile to shift downwards by 0.445 in log_10_ *J* for each increase in *β* by 0.1, or equivalently log_10_ *J* increases by about 0.1 for each 1 nm increase in the (curvature-free) persistence length.

#### Tandem effect of stiffness and bend

Since the main effect of bend angle and overall stiffness appears to be an overall shift in the cyclization profile, that would seem to imply the existence of families of molecules with quite different mixes of stiffness and bend but very similar cyclization profiles. For example, if we combine the regression fits (2) and (3) by assuming the effects are additive (and averaging the two very similar constant terms), we have:

4 $$a\approx 1.325+0.021({\rm{Bend}}\,{\rm{angle}}-90)-4.45(\beta -1)\mathrm{.}$$

In this case, we would expect very similar cyclization profiles if

5 $$0.021({\rm{Bendangle}}-90)-4.45(\beta -1)\mathrm{=0}$$

or

6 $${\rm{Bend}}\,{\rm{angle}}=90+212(\beta -1)\mathrm{.}$$

**Figure 4:**
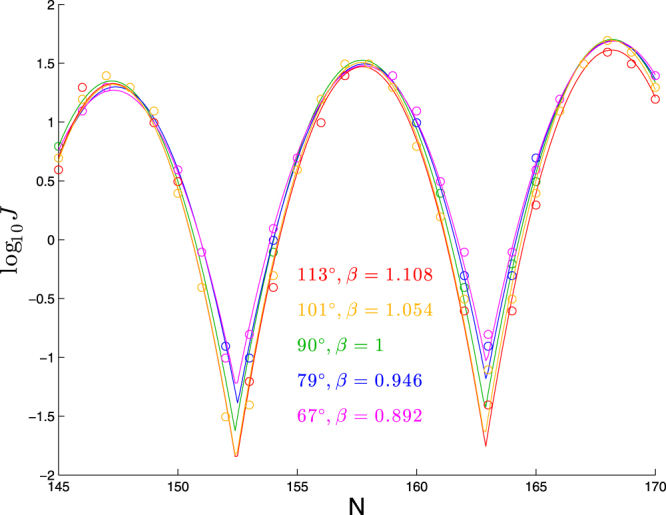
Cyclization profiles for five pairs (Bendangle, *β*) satisfying (6). Each molecule consists of a planar bend over 63 bp followed by a straight segment of *N* − 63 bp.

To check this hypothesis, we show in Fig. 4 cyclization profiles for five pairs of (Bendangle,*β*) satisfying (6). Indeed we can see the five curves agree quite well, especially in the regions surrounding the peaks. There remains some variation in the troughs, consistent with the dependence of the parameter *c* on *β* seen in Table 3. However, note that detecting these differences experimentally would be challenging, as they represent poor cyclizers for which *J* is difficult to measure with high precision.

### Intrinsic twist has a distinctive effect on results, but non-planar bending could mask that effect

#### Effect of helical repeat/intrinsic twist

**Figure 5:**
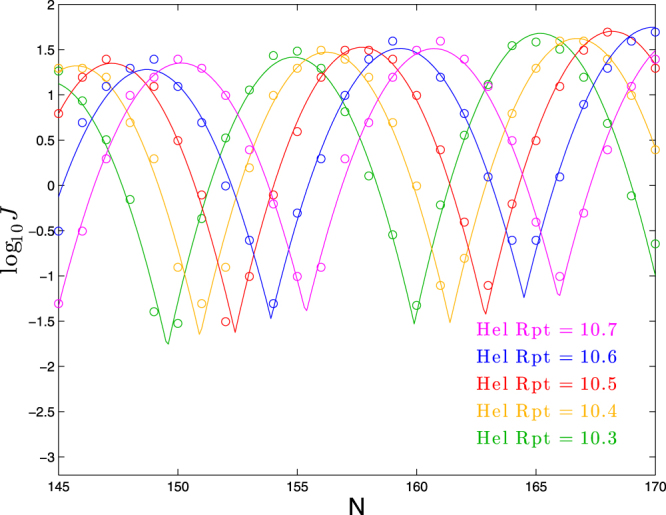
Dependence of cyclization profile on intrinsic twist (expressed in terms of helical repeat, the number of basepairs in which a full twist occurs). Each molecule consists of a planar bend of 90° over 63 bp followed by a straight segment of *N* − 63 bp.

We show in Fig. 5 the effect of varying the intrinsic twist within a molecule consisting of a 63-bp planar bend followed by a straight segment. By eye, the main effect is a horizontal shift with increasing helical repeat (decreasing intrinsic twist). This is confirmed by fits to our functional form, as seen in Table 4, where we see a clear upward trend in *ϕ* with bend angle. We also see from the fits a variation in the period parameter *p* that roughly mirrors the helical repeat, as we would expect (but which is more difficult to detect from the plots given just two periods’ worth of data). The other three parameters (*a*, *b*, and *c*) do not appear to show any consistent trend.

**Table 4:** Best-fit parameters for each curve in Fig. 5 to log_10_ *J* = *a* + *b*(*N* − *ϕ*) + *c*[*f*(*N*, *ϕ*, *p*)]^2^.

Helical repeat	*ϕ*	*p*	*a*	*b*	*c*
10.3	144.40	10.33	1.15	0.025	−0.112
10.4	145.68	10.48	1.32	0.014	−0.112
10.5	147.16	10.48	1.35	0.017	−0.112
10.6	148.60	10.59	1.28	0.022	−0.102
10.7	150.07	10.59	1.35	0.015	−0.102

Applying linear regression to the best-fit values of *ϕ*, we find:

7 $$\varphi \approx 14.3({\rm{helical}}\,{\rm{repeat}}-10.5)+147.2$$

Thus, in this range of helical repeat, the primary effect of increasing helical repeat is to shift the cyclization profile rightward by 1.43 bp for each 0.1 of helical repeat. This result matches the intuitive prediction that the profile would have *ϕ*, the location of the first peak (in this window of *N*) occur at 14 times the helical repeat.

#### Effect of helicity in bending

Next we will explore the effect of non-planarity of an intrinsic bend on the cyclization profile, specifically for the case where the intrinsic shape is helical for the first 63 bp and straight thereafter. We fix the bend angle per bp within the helical section at (*π*/2)/63 (so that the total effective magnitude of bending is 90°) and use the ratio of pitch-to-circumference to categorize the extent of helicity. Following the description of intrinsic shape from Methods, for a given pitch-to-circumference ratio, we solve Eqs (27) and (28) for *α* and *ψ*, and those two parameters together with *β* = 2*π*/10.5 determine the intrinsic shape parameters ${\hat{\theta }}_{j}^{i}$ and ${\hat{a}}_{j}^{i}$ of the helix.

**Figure 6:**
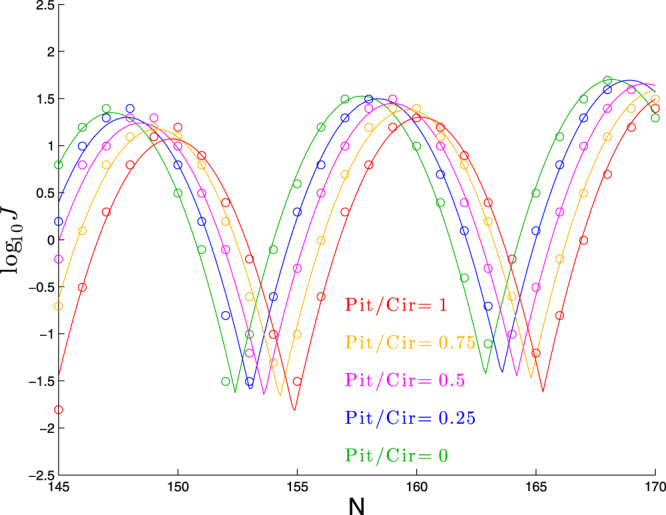
Dependence of cyclization profile on the ratio of pitch to circumference. Each molecule consists of a left-handed helical bend of 90° over 63 bp followed by a straight segment of *N* − 63 bp.

We show in Fig. 6 the effect of changing helicity in this way. As seen in this figure, helicity produces a horizontal shift in the cyclization profile, which is reflected in the best-fit parameter values in Table 5 by the clear upward trend in the phase-shift parameter *ϕ*. In addition, increasing helicity pulls the cyclization profile downward, as reflected by the downward trend in the parameter *a*. The other three parameters appear to be fairly constant, except perhaps for a mild upward trend in the parameter *b*. Applying linear regression to the best-fit values of *ϕ* and *a*, we find:

8 $$\varphi \approx 147.15+2.49({\rm{Pitch}}/{\rm{Circumf}}),\quad a\approx 1.36-0.27({\rm{Pitch}}/{\rm{Circumf}})\quad (\mathrm{for}\,\mathrm{Pitch} > 0)$$

**Table 5:** Best-fit parameters for each curve in Fig. 6 to log_10_ *J* = *a* + *b*(*N* − *ϕ*) + *c*[*f*(*N*, *ϕ*, *p*)]^2^.

Pitch/Circumf	*ϕ*	*p*	*a*	*b*	*c*
0	147.16	10.48	1.35	0.017	−0.112
0.25	147.79	10.52	1.30	0.019	−0.110
0.5	148.33	10.57	1.24	0.020	−0.108
0.75	149.02	10.52	1.18	0.020	−0.107
1	149.65	10.43	1.07	0.022	−0.112

Thus, for each increase in Pitch/Circumf by 0.1 we expect the cyclization profile to shift right by 0.25 bp and downward by 0.03 in log_10_ *J*. Presumably this means that once Pitch/Circumf is 0.4 or larger, it might plausibly be detected as a shift by a single bp in the cyclization profile (and it would seem to take about that same amount of helicity in order for the downward shift in the profile to be detectable).

**Figure 7:**
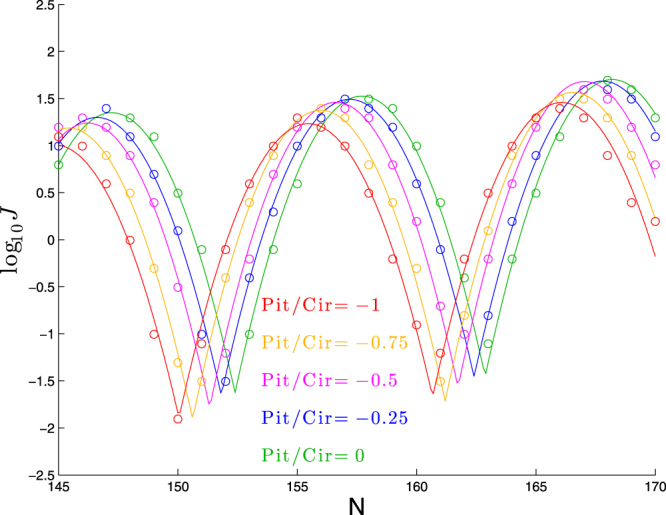
Dependence of cyclization profile on the ratio of pitch to circumference. Each molecule consists of a right-handed helical bend of 90° over 63 bp followed by a straight segment of *N* − 63 bp.

Next we confirm in Fig. 7 a similar effect for helices of the opposite handedness, in which the horizontal shift is leftward while the vertical shift remains downward. These results are confirmed by the best-fit parameters in Table 6. Applying linear regression to the best-fit values of *ϕ* and *a*, we find:

9 $$\varphi \approx 147.19+2.42({\rm{Pitch}}/{\rm{Circumf}}),\quad a\approx 1.37+0.32({\rm{Pitch}}/{\rm{Circumf}}),\quad (\mathrm{for}\,\mathrm{Pitch} < 0)$$

very similar formulae as in the positive helicity case, except for the sign-change in the linear coefficient for the formula for *a* (since *a* decreases as helicity deviates from zero in either direction). It thus would be sensible to collapse the two relationships into one (making the *ad hoc* choice to average the coefficients):

10 $$\varphi \approx 147.17+\mathrm{2.45(}{\rm{Pitch}}/{\rm{Circumf}}),\quad a\approx 1.37-\mathrm{0.3|}{\rm{Pitch}}/{\rm{Circumf}}\mathrm{|.}$$

**Table 6:** Best-fit parameters for each curve in Fig. 7 to log_10_ *J* = *a* + *b*(*N* − *ϕ*) + *c*[*f*(*N*, *ϕ*, *p*)]^2^.

Pitch/Circumf	*ϕ*	*p*	*a*	*b*	*c*
0	147.16	10.48	1.35	0.017	−0.112
−0.25	146.53	10.59	1.30	0.018	−0.109
−0.5	146.13	10.40	1.24	0.021	−0.116
−0.75	145.33	10.58	1.19	0.018	−0.114
−1	144.74	10.62	1.01	0.021	−0.107

#### Tandem effect of intrinsic twist, helicity, and bend angle

To test the last two sets of results, we look for a family of molecules with quite different mixes of values for intrinsic twist, helicity, and bend angle but identical cyclization profiles (or nearly so). We found that both intrinsic twist and helicity change the phase parameter *ϕ*, so that (assuming their two regression equations can be combined):

11 $$\varphi =147.2+14.3({\rm{helical}}\,{\rm{repeat}}-10.5)+\mathrm{2.45(}{\rm{Pitch}}/{\rm{Circumf}}\mathrm{).}$$

Thus we conclude that to maintain the same value of *ϕ* if we change the helical repeat, we need

12 $${\rm{Pitch}}/{\rm{Circumf}}=-5.84({\rm{helical}}\,{\rm{repeat}}-10.5)$$

However, if we choose that ratio of pitch-to-circumference, that will lower the height parameter *a*, so we compensate for that by adapting bend angle; to determine what value of bend angle to use we again consult the relevant regression equations: since each unit of |Pitch/Circumf| decreases *a* by about 0.3, and each degree of bend angle above 90 increases *a* by about 0.021, we want

13 $${\rm{Bend}}\,{\rm{angle}}=90+\frac{0.3}{0.021}|{\rm{Pitch}}/{\rm{Circumf}}|=90+\mathrm{14.3|}{\rm{Pitch}}/{\rm{Circumf}}\mathrm{|.}$$

**Figure 8:**
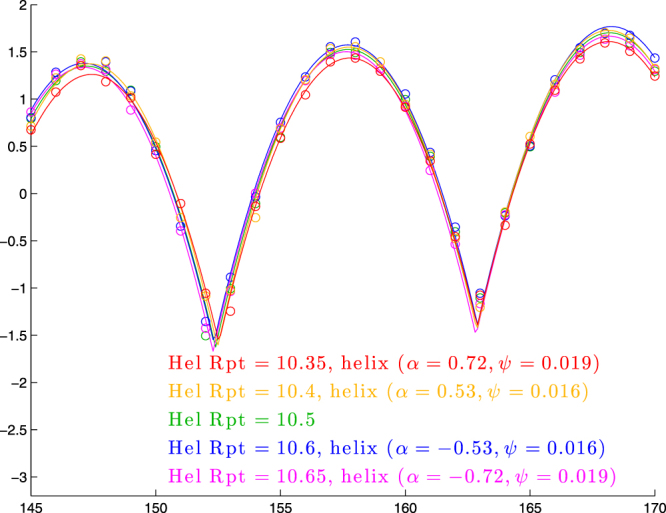
Cyclization profiles for five molecules in which bend-angle and helicity are varied to counteract changes in helical repeat. For helical repeats 10.4 and 10.6, we use Pitch/Circumf = ±0.584 according to (12) and bend angle of 90 + 14.3(0.584) = 98.4 degrees according to (13). Similarly, for helical repeats 10.35 and 10.65, we use Pitch/Circumf = ±0.876 and bend angle 90 + 14.3(0.876) = 102.5. In each case, the values of Pitch/Circumf and bend angle determine helical parameters *α* and *ψ* shown in the legend, according to (26) and (27).

We show in Fig. 8 a set of cyclization profiles that varies helical repeat, bend-angle, and helicity in tandem according to (12) and (13).

We see that the five profiles line up quite closely, though the most extreme values of helical repeat (10.35 and 10.65) show some signs of being lower than the rest. Indeed, for helical repeats 10.3 and 10.7, the downward deviation becomes quite significant (results not shown), consistent with our prior result that the dependence of *a* on Pitch/Circumf breaks down for |Pitch/Circumf| > 1 (for helical repeats 10.3 and 10.7, the value of |Pitch/Circumf| from (12) would be 1.168).

### Twist-to-bend stiffness affects the peak-to-trough difference

**Figure 9:**
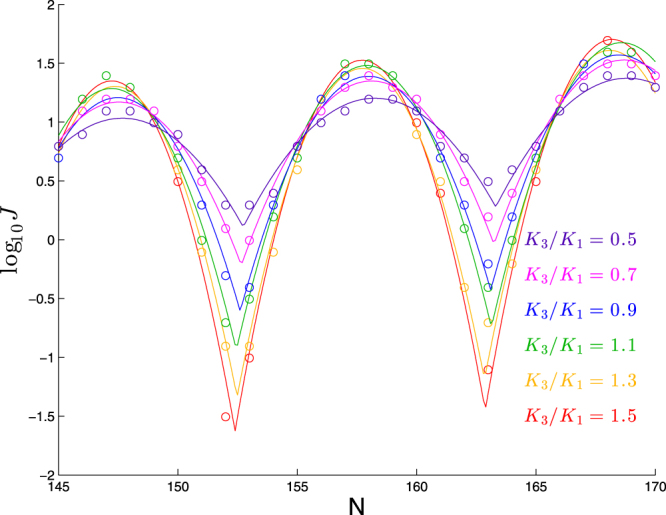
Dependence of cyclization profile on *K*_3_/*K*_1_. Each molecule consists of a planar bend of 90° over 63 bp followed by a straight segment of *N* − 63 bp. The stiffness *K*_3_ is varied while the remaining five stiffnesses are held fixed.

We show in Fig. 9 the effect of scaling only *K*_3_. As seen in this figure, and in the best-fit parameter values in Table 7, the effect of this change is quite different than the effects seen in previous plots: we see a substantial change in the *shape* of the plot, as reflected in the quadratic coefficient *c* becoming more negative as *K*_3_/*K*_1_ increases, consistent with the intuition that larger *K*_3_/*K*_1_ values lead to deeper troughs in the cyclization profile, since the trough configurations involve significant twisting. At the same time, there is also an overall upward shift in the peak heights with *K*_3_/*K*_1_, as reflected in the upward trend in the parameter *a*. This shift is counterintuitive, since an increase in *K*_3_/*K*_1_ increases the twist term in the energy and leaves the remaining terms constant, and yet this leads to a larger *J*-factor (at least at the peaks), which suggests a *lower* energy. The other three parameters (*ϕ*, *p*, and *b*) appear to be fairly constant. Applying linear regression to the best-fit values of *c* and *a*, we find:

14 $$c\approx -0.114-0.077({K}_{3}/{K}_{1}-1.5),\quad a\approx 1.37+0.30({K}_{3}/{K}_{1}-1.5)$$

**Table 7:** Best-fit parameters for each curve in Fig. 9 to log_10_ *J* = *a + b*(*N* − *ϕ*) + *c*[*f*(*N*, *ϕ*, *p*)]^2^.

*K*_3_/*K*_1_	*ϕ*	*p*	*a*	*b*	*c*
0.5	147.45	10.58	1.03	0.016	−0.036
0.7	147.37	10.58	1.17	0.017	−0.053
0.9	147.37	10.49	1.21	0.017	−0.069
1.1	147.11	10.68	1.28	0.018	−0.081
1.3	147.30	10.36	1.30	0.015	−0.101
1.5	147.16	10.48	1.35	0.017	−0.112

Thus, in this range of *K*_3_/*K*_1_, the quadratic coefficient decreases by about 0.0077 for each increase in *K*_3_/*K*_1_ by 0.1. In terms of the profile itself, note that the quadratic term ranges from 0 (at *n* = *ϕ* modulo *p*) to −*c*(*p*/2)^2^ ≈ −*c*(10.5/2)^2^ = −27.6*c*, so that the peak-to-trough height difference (in log_10_ *J*) increases by about 27.6 × 0.0077 = 0.21 for each increase in *K*_3_/*K*_1_ by 0.1.

**Figure 10:**
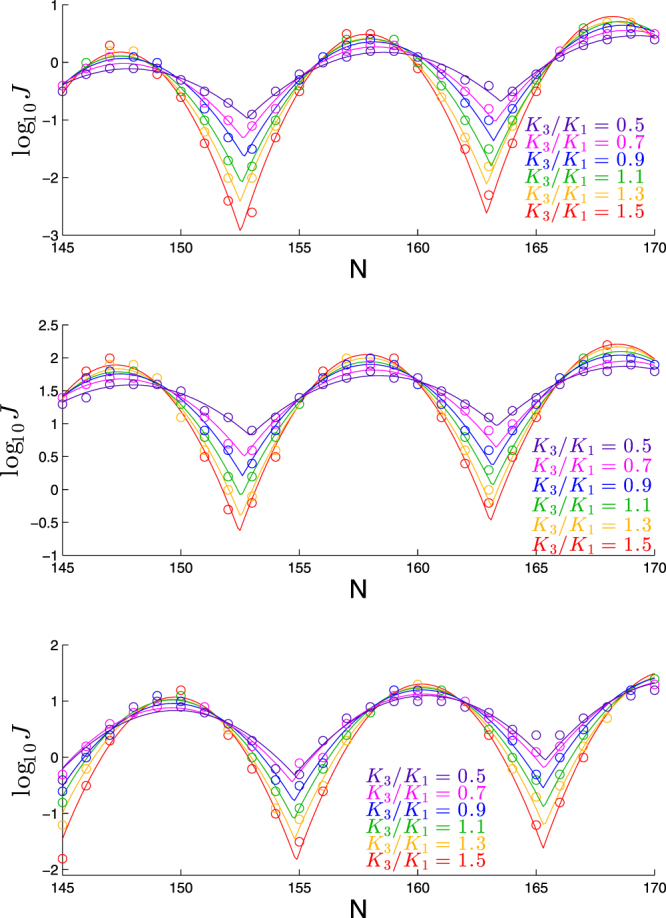
Dependence of cyclization profile on *K*_3_/*K*_1_ for variants of molecule in Fig. 9 bend angle of 45° (first panel); reduced stiffness corresponding to *β* = 0.87 (second panel); helicity corresponding to Pitch/Circumf = 1 (third panel).

This effect is fairly consistent across the range of elastic rods studied here. For example, we show in Fig. 10 the effect of varying *K*_3_/*K*_1_ for three other elastic rods. Qualitatively we see the same trend as we change *K*_3_/*K*_1_, though the absolute scale of log_10_ *J* is different for each case (as we expect from studying these cases earlier).

**Figure 11:**
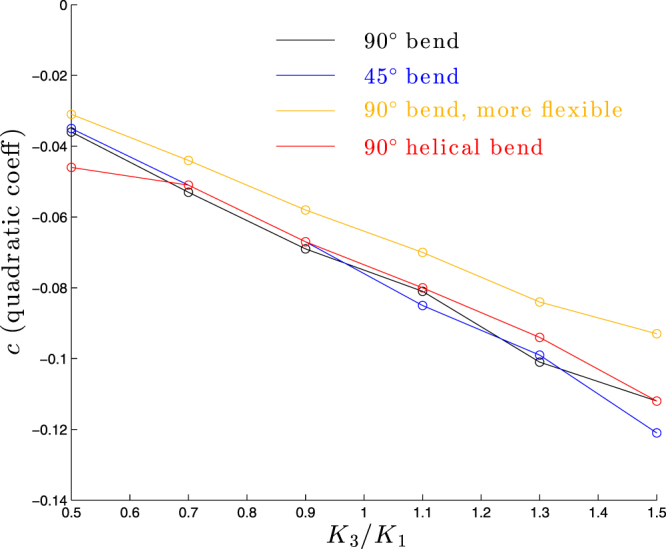
Dependence of quadratic coefficient *c* on *K*_3_/*K*_1_ for molecules considered in Figs 9 and 10.

To make a more quantitative comparison, we show in Fig. 11 a plot of the quadratic coefficient *c* versus *K*_3_/*K*_1_ for all the molecules studied and see quite similar results for all cases.

### More intricate bending patterns further muddy the waters

**Figure 12:**
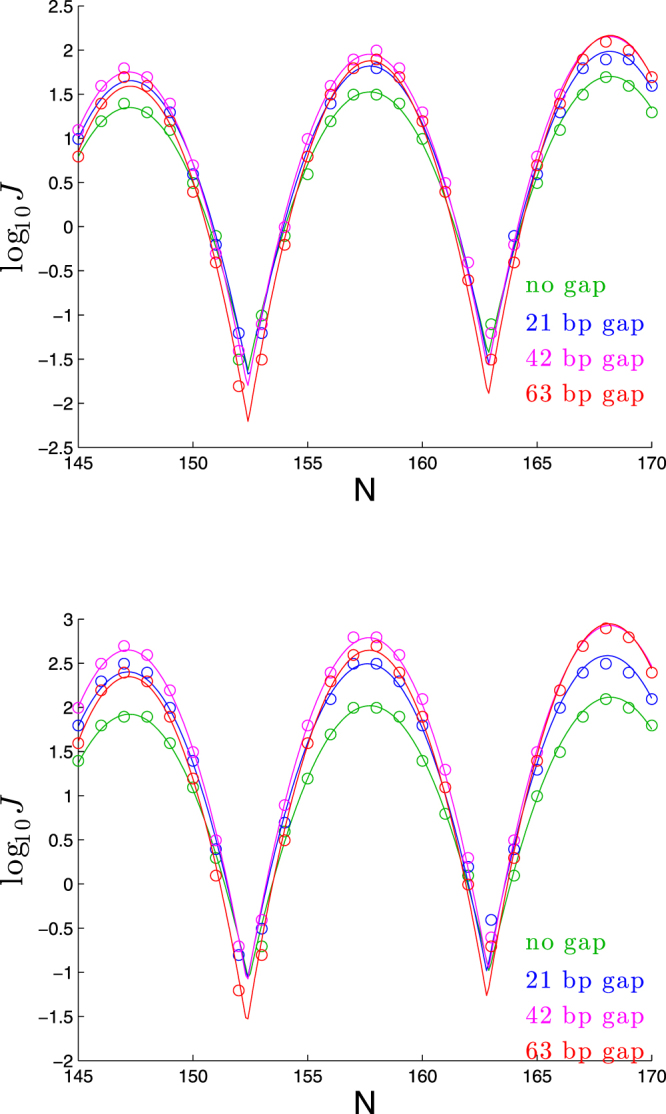
Dependence of cyclization profile on gap between two planar bends. In the first panel, the DNA has a 60° bend in the first 42 bp, followed by a straight segment of length 0, 21, 42, or 63 bp, followed by a 30° bend in the next 21 bp, and the rest of the DNA is straight. The setup for the second panel is the same, except that the first bend is 80° and the second bend is 40°.

Of course in a real DNA molecule, intrinsic bending will not occur at a single location. As we show in Fig. 12, when we insert a straight segment between two planar bends, the results are harder to categorize than the consistent trends found in our earlier analyses. In general, increasing the spread between the two bends leads to higher peaks and lower troughs, but not in all cases: for large enough gaps, the peak heights decrease (e.g., note the left-most peaks in each plot, in which the 42 bp gap yields a higher peak than either a 21 bp gap or a 63 bp gap). See Supplementary Information for a figure showing the effect of separating two bends out-of-phase.

**Figure 13:**
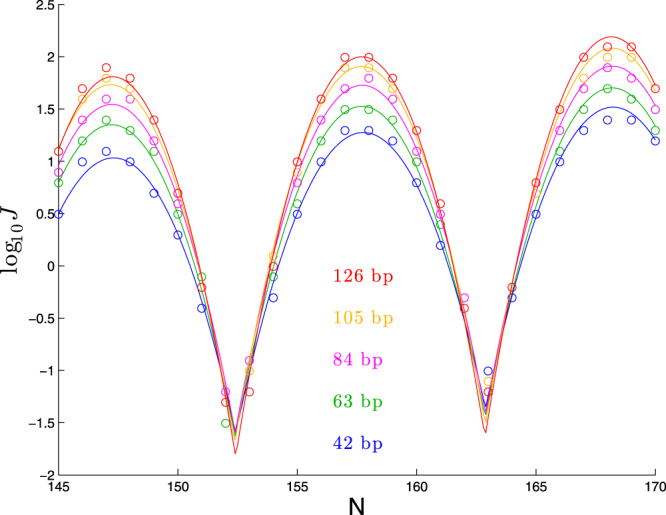
Dependence of cyclization profile on number of bp over which 90° of bend occurs.

Even with a single bend, spreading the bend out over more basepairs has a substantial effect on the peak heights, as seen in Fig. 13.

The quantitative results of our earlier analyses fixed the bending to occur in the first 63 bp, and even in that very special case, we found that different parameters of the DNA had very similar effects on the cyclization profiles, such that it was hard to envision extracting values for those parameters from cyclization experiments, with the exception, perhaps, of the value of *K*_3_/*K*_1_ being uniquely related to the peak-to-trough difference. The results in this section make the prospects of parameter-extraction all the more daunting, since bend-separation has a substantial impact on peak heights. Furthermore, the length of a bend in bp has a clear impact on peak-to-trough difference, calling into question the ability to determine *K*_3_/*K*_1_ from a cyclization profile.

## Discussion

Although many of the results discussed here are negative, in the sense of pointing to an inability to solve the inverse problem of finding DNA parameters from cyclization results, there are nevertheless also positive messages to take from the results.

If we could determine some DNA parameters by means independent of cyclization, such as by a reliable DNA model, then the results shown here could possibly be used to estimate other DNA parameters with some confidence. For example, if the DNA intrinsic shape is known fairly precisely, then the peak locations, heights, and troughs could be used to estimate the DNA stiffnesses.

In a similar vein, for DNA models with many parameters^16,17^, the results here can be used as a way of interpreting that large set of parameters. Specifically, though the intrinsic shape predicted by such a model can be visualized, it can be harder to make sense of the “stiffness” of a DNA segment when the stiffness is described by hundreds or thousands of sequence-dependent local stiffness parameters. In that situation, performing computations of the style done here can allow one to “calibrate” a many-parameter model by finding which very-simple elastic-rod gives a cyclization profile that matches the prediction of the more complicated model.

**Figure 14:**
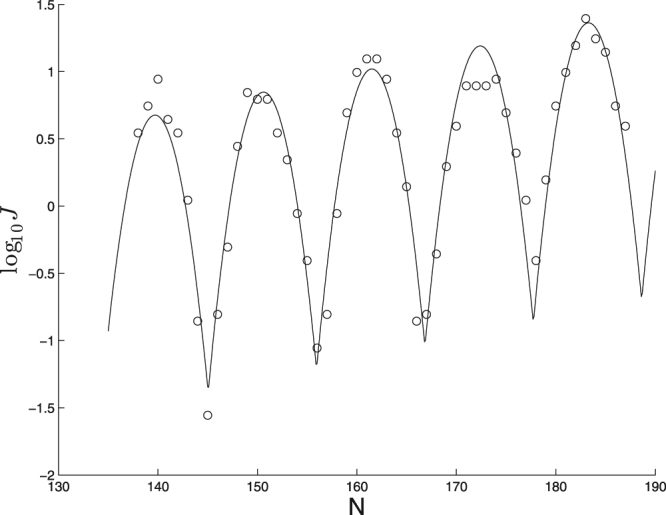
Cyclization profile (circles), and best fit functional form (line) for a sequence involving a run of A-tracts.

As a proof-of-principle demonstration of this idea, consider the results shown in Fig. 14 showing Monte Carlo estimates of *J* computed within the cgDNA model^17^. This model takes as input the *N*-basepair DNA sequence and outputs an intrinsic shape within a rigid-base model and a (12*N* − 6) × (12*N* − 6) stiffness matrix, whose nonzero entries involve dense 18 × 18 blocks that overlap in 6 × 6 blocks. With so many distinct values in the stiffness matrix, it is very hard to read from it an overall intuitive sense of “stiffness”. We can get at least a primitive sense of that stiffness by a calibration to the simplistic model in this article. In this case, we will use just the values *c* ≈ −0.0723 and *a* ≈ 0.847 that come from fitting the Monte Carlo results to the functional form (29).

First we note that the first part of Eq. (14) indicates that *c* ≈ −0.0723 corresponds to *K*_3_/*K*_1_ ≈ 1.0.

Next we seek to estimate the overall stiffness parameter *β*. In light of the degeneracy highlighted in this article, we know the *J*-factor results are unlikely to allow us to extract both intrinsic bending and stiffness, so we first estimate intrinsic bend from the cgDNA model independent of the *J*-factor computations: the dot product (**r**_145_ − **r**_125_) ⋅ (**r**_21_ − **r**_1_) yields an estimate of 100° (such substantial intrinsic bending is not surprising for this particular molecule, as it contains a central segment of A-tracts). Inserting this intrinsic bend, and *a* ≈ 0.847, into Eq. (4), we estimate *β* ≈ 1.15. (We could refine this estimate by noting that from the second part of Eq. (14), the estimate *K*_3_/*K*_1_ ≈ 1.0 suggests a shift in the baseline value of *a* by 0.3(1.0–1.5) = −0.15, and then applying that shift to the value 1.325 in Eq. (4) yields a new estimate *β* ≈ 1.12.)

These computations, though admittedly primitive, nevertheless do provide some intuition by condensing the many parameters in the stiffness matrix to the two easily-interpretable parameters *β* and *K*_3_/*K*_1_. Furthermore, a fit to the simplistic model may highlight interesting features in more realistic models, e.g., in Fig. 14 we can see substantial flattening in the fourth peak that is not replicated in any computations within the simplistic model, and hence appear to be a distinctive feature of the combination of this particular molecule and this particular DNA model. One might expect to derive similar insights if one applies our proposed calibration to other feasible experimental designs, such as single-basepair substitution.

## Methods

### Models for DNA configuration and energy

We treat the DNA using a rigid basepair model that assigns to each basepair a rigid frame, numbered by an index *i* running from 1 to *N* (the number of basepairs). Each frame has an “origin” **r**^*i*^ and a set of three orthonormal “directors” ${{\bf{d}}}_{j}^{i}$ (*j* = 1, 2, 3), which we will sometimes collect into an orthogonal matrix $${R}^{i}\equiv [\begin{array}{ccc}{{\bf{d}}}_{1}^{i} & {{\bf{d}}}_{2}^{i} & {{\bf{d}}}_{3}^{i}\end{array}]$$. We will choose coordinates so that frame *i* = 1 has origin *r* = 0 and standard orientation *R*^1^ = *I*, the 3 × 3 identity matrix.

The relative translation and rotation between frames *i* and *i* + 1 are described by “internal coordinates”: rotation angles *θ*^*i*^ (roll, tilt, twist) and translations **a**^*i*^ (shift, slide, rise). The definitions of *θ*^*i*^ and **a**^*i*^ follow Lankaš *et al*.^18^, which defines *θ*^*i*^ as Cayley angles of the relative rotation, and *a*^*i*^ as the translation vector in the coordinate system of the “midframe” between frame *i* and *i* + 1. These choices guarantee the six quantities transform simply should we decide to number the basepairs in the opposite order (5′-3′ versus 3′-5′). For small angles, the Cayley angles are close to the Euler angles sometimes used to define roll, tilt, and twist.

To include the possibility of extensibility/shearability, we assume an energy function

15 $$E=\frac{1}{2}\sum _{i=1}^{N}\sum _{j=1}^{3}[{K}_{j}{({\theta }_{j}^{i}-{\hat{\theta }}_{j}^{i})}^{2}+{A}_{j}{({a}_{j}^{i}-{\hat{a}}_{j}^{i})}^{2}].$$

Thus, the energy is quadratic in the internal coordinates, with six stiffness parameters *K*_1_, *K*_2_, *K*_3_, *A*_1_, *A*_2_, *A*_3_ (hence, no cross-terms like roll-twist coupling and no sequence dependent stiffnesses). Our computations explore the effects of varying the bending stiffness *K*_1_, always with *K*_2_ = *K*_1_, corresponding to an isotropic rod, a commonly used assumption for modeling DNA on a length scale longer than a few helical turns, relying on the idea that local anisotropies (such as the distinction between major and minor groove bending) would be averaged out by the helical twist (see, e.g., Kehrbaum and Maddocks^19^). In the Supplementary Information, we document that this assumption is reasonable, by showing that *J*-factors change negligibly for 1 ≤ *K*_2_/*K*_1_ ≤ 8 as long as we maintain a constant harmonic average of *K*_1_, *K*_2_.

Our baseline value of *K*_1_ is (46.3/0.34)*RT*, corresponding to a persistence length (in the absence of intrinsic bending) of 46.3 nm, and we vary *K*_1_ to cover a persistence-length range of 40.3–52.3 nm. Similarly, we vary the twist-to-bend-stiffness ratio *K*_3_/*K*_1_ over the range 0.5–1.5. For the shear and stretch stiffnesses *A*_*j*_, we use values (*A*_1_, *A*_2_, *A*_3_) = (14, 24, 85) in units of *RT*/*Å*, preliminary estimates derived from molecular dynamics simulations of DNA (D. Petkevičiūtė, private communication); in the Supplementary Material, we show that removing the three *A*_*i*_ in favor of an inextensible-unshearable model has no significant impact on the results.

The quantities ${\hat{\theta }}_{j}^{i}$ and ${\hat{a}}_{j}^{i}$ are the internal coordinates for the minimum-energy configuration of the DNA, which we take to be made up of helical arcs, circular arcs, or straight segments, all with constant twist.

For a helical segment we use

16 $${\hat{a}}_{1}^{i}=-\frac{\ell }{D}\,\cos \,\alpha \,\sin \,\psi \,\sin \,(\beta /2)\,\sin \,((2i-1)\beta /2)[1+\,\tan \,(\psi /2)\,\tan \,(\beta /4)\,\sin \,\alpha ]$$

17 $${\hat{a}}_{2}^{i}=-\frac{\ell }{D}\,\cos \,\alpha \,\,\sin \,\psi \,\sin \,(\beta \mathrm{/2)}\,\cos \,\mathrm{((2}i-\mathrm{1)}\beta \mathrm{/2)}[1+\,\tan \,(\psi \mathrm{/2)}\,\tan \,(\beta \mathrm{/4)}\,\sin \,\alpha ]$$

18 $${\hat{a}}_{3}^{i}=\frac{\ell }{D}{\cos }^{2}\alpha (\cos \,\,\psi +\,\cos \,(\beta \mathrm{/2))}+\frac{\ell }{D}\,\sin \,\alpha [\sin \,\psi \,\sin \,(\beta \mathrm{/2)}+\,\sin \,\alpha +\,\sin \,\alpha \,\cos \,\,\psi \,\cos (\beta \mathrm{/2)}]$$

19 $$(D\equiv 1+\,\cos \,\psi \,\cos \,(\beta \mathrm{/2)}+\,\sin \,\psi \,\sin \,(\beta \mathrm{/2)}\,\sin \,\,\alpha ),$$

and

20 $${\hat{\theta }}_{1}^{i}=-\frac{2}{D-1}\,\sin \,\psi \,\sin \,\mathrm{((2}i-\mathrm{1)}\beta \mathrm{/2)}\,\cos \,\,\alpha $$

21 $${\hat{\theta }}_{2}^{i}=-\frac{2}{D-1}\,\sin \,\psi \,\cos \,\mathrm{((2}i-\mathrm{1)}\beta \mathrm{/2)}\,\cos \,\alpha $$

22 $${\hat{\theta }}_{3}^{i}=\frac{2}{D-1}(\cos \,\psi \,\sin \,(\beta \mathrm{/2)}-\,\sin \,\psi \,\cos \,(\beta \mathrm{/2)}\,\sin \,\alpha ),$$

with the parameters *ψ*, *β*, *α* controlling (respectively) the intrinsic bending, intrinsic twisting, and helicity, as spelled out below, and $\ell$ controlling the basepair spacing (we take $\ell =0.34$ nm). Given that we chose coordinates so that frame *i* = 1 has origin *r* = 0 and standard orientation *d*_*j*_ = *e*_*j*_, these internal coordinates yield a sequence of frames whose origins are:

23 $$x=-\frac{\ell }{2}\frac{\cos \,\alpha }{\sin \,\psi }+\frac{\ell }{2}\frac{\cos \,\alpha }{\sin \,\psi }\,\cos \,\,\mathrm{(2(}i-\mathrm{1)}\psi )$$

24 $$y=(i-\mathrm{1)}\ell \,\sin \,\alpha $$

25 $$z=\frac{\ell }{2}\frac{\cos \,\alpha }{\sin \,\psi }\,\sin \,\mathrm{(2(}i-\mathrm{1)}\psi ),$$

all of which lie on a helix with helical axis parallel to the *y*-axis and passing through the point $(-\frac{\ell }{2}\frac{\cos \,\alpha }{\sin \,\psi },0,0)$. The directors ${{\bf{d}}}_{3}^{i}$ are all tangent to this helix, so that

26 $$\cos \,({\rm{B}}{\rm{e}}{\rm{n}}{\rm{d}}\,{\rm{a}}{\rm{n}}{\rm{g}}{\rm{l}}{\rm{e}}\,{\rm{p}}{\rm{e}}{\rm{r}}\,{\rm{b}}{\rm{p}})={{\bf{d}}}_{3}^{i+1}\cdot {{\bf{d}}}_{3}^{i}={\cos }^{2}\,\alpha \,\cos \,(2\psi )+{\sin }^{2}\,\alpha .$$

We will quantify the nonplanarity of the bend by the geometric property of the helix:

27 $$\frac{{\rm{Pitch}}}{{\rm{Circumference}}}=\,\tan \,\,\alpha \frac{\sin \,\psi }{\psi }\mathrm{.}$$

(Note that if *ψ* and *α* are small, then the bend angle per bp is approximately 2*ψ* and the ratio of pitch to circumference is approximately *tanα*.) The parameter *β* controls the intrinsic twist, in the sense that the directors ${{\bf{d}}}_{1}^{i},{{\bf{d}}}_{2}^{i}$ are rotated by angle *iβ* relative to the Serret-Frenet normal vector of the helix.

Setting *α* = 0 yields a circular arc, and setting *α* = *ψ* = 0 yields a straight segment.

#### Relation of *J*-factor to probability density

The *J*-factor is traditionally described as the concentration of one end of the DNA in the neighborhood of the other end, and indeed the *J* factor is reported in units of concentration, most often nM. But this “concentration” is a bit subtle, as it must account for the orientation of the end of the DNA, since the orientation significantly affects the ability of the DNA to form a cyclized product. Mathematically, we can capture this combination of effects by considering the probability density function for frame #*N*; this probability density is with respect to the space ${{\mathbb{R}}}^{3}\times SO(3)$ where *SO*(3) is the set of orthogonal 3-by-3 matrices with determinant 1.

Because of helical structure of DNA, in a cyclized configuration, frame #*N* of the last basepair would be located at position $\mathrm{(0,}\,\mathrm{0,}-\ell )$, and be oriented with its tangent director ${{\bf{d}}}_{3}^{N}$ roughly vertical, and its other directors ${{\bf{d}}}_{1}^{N},{{\bf{d}}}_{2}^{N}$ twisted roughly 2*π*/(10.5) short of **e**_1_, **e**_2_. To avoid these complexities, we adopt a strategy commonly seen in the literature^14^ to append a fictitious frame #(*N* + 1) to the elastic rod and describe cyclization as the coincidence of frames #1 and #(*N* + 1). In this case, the *J*-factor is the value of the probability density function at the point $(0,I)\in {{\mathbb{R}}}^{3}\times SO(3)$.

Such a probability density function can only be made sensible if one has defined a measure on ${{\mathbb{R}}}^{3}\times SO(3)$. For the ${{\mathbb{R}}}^{3}$ portion, the volume is a natural choice for measure, and that choice makes *J* have the desired units of concentration. For the *SO*(3) portion, the question is more subtle. In a seminal paper in cyclization, Flory^11^ accounts for this effect by expressing the free energy of cyclization in terms of two probability densities, one for the angle between the tangent vectors of the two ends and one for the torsional angle between the two ends, and other more recent studies such as Levene and Crothers^9^ and Czapla, Swigon, and Olson^14^ have adopted this approach.

However, defining a torsion angle when the tangent vectors are not aligned requires some arbitrary choices akin to the variety of conventions for defining Euler angles, and for larger angles, the differences between conventions become larger. We adopt a different approach, choosing as our measure on *SO*(3) the unique Haar measure that is invariant under rotation (multiplication of the entire group by any particular element of *SO*(3)). This gives a unique result even for large angles, and, as we argue below, matches the standard Flory approach for small angles.

### Monte Carlo implementation

We simulate cyclization by Monte Carlo, in which a large ensemble (10^13^–10^16^) of random configurations is generated and the concentration *J* estimated by choosing a small region in ${{\mathbb{R}}}^{3}\times SO(3)$ containing the cyclization point (**0**, ***I***) and dividing the number of simulated molecules whose far end lands in this region by the volume of this region.

In order to generate the random molecules, we use an implementation of the “half-molecule” technique as developed by Alexandrowicz *et al*.^15^ and subsequently used for DNA Monte Carlo simulations by Levene and Crothers^9^ and Czapla, Swigon and Olson^14^. In this technique, one computes *M* random instances each of the first and second halves of the DNA and then considers all first-half-second-half pairs in order to generate *M*^2^ random molecules. The time savings of this method allows us to generate the large sample sizes required to obtain estimates of *J* to our desired accuracy of 0.1 in ${\mathrm{log}}_{10}\,J$ (for the poorest cyclizers, we use *M* = 2^26^–2^28^ to generate 10^15^–10^17^ molecules; for the best cyclizers, *M* = 2^23^ suffices).

Our implementation follows closely the “cube-binning” idea presented in Czapla, Swigon, and Olson^14^ in which half-molecules are sorted into bins according to end-location for second-halves, and a rotated end-location for first-halves (**r**_*m*:*N*+1_ and $-{T}_{\mathrm{1:}m}^{-1}{{\bf{r}}}_{\mathrm{1:}m}$ in the notation of Czapla, Swigon, and Olson^14^), so that pairs need only be computed if the bins of the first-half and second-half are sufficiently close to each other.

In terms of end-to-end distance, our closure condition involves the natural choice of defining a tolerance *ε* on the Euclidean distance between **r**_*m*:*N*+1_ and $-{T}_{\mathrm{1:}m}^{-1}{{\bf{r}}}_{\mathrm{1:}m}$, with corresponding volume $\frac{4}{3}\pi {\varepsilon }^{3}$. However, parallel to the discussion in the previous section, our closure conditions differ from the standard Flory approach to the orientation aspect of “closure”: rather than assign two tolerances to the angle between the terminal base pair *d*_3_ directors and the torsion angle, we define a single tolerance *δ* on a natural “distance” in *SO*(3) between frames 1 and *N* + 1. Details of this distance can be found in the Supplementary Information, including the result that the “ball of radius *δ*” using this distance has volume $\frac{2}{\pi }(\arcsin \,\delta -\delta \sqrt{1-{\delta }^{2}})$ (using the normalized Haar measure on *SO*(3)).

We note that for the treatment of *SO*(3) used in Czapla, Swigon, and Olson^14^ (“closure” defined by **d**_3_ angle < arccos(1 − *ν*_*ε*_) and |**d**_1_ angle| < *τ*_*ε*_, with arccos(1 − *ν*_*ε*_) = *τ*_*ε*_), the two approaches agree in the limit of small tolerances, in the sense that the Haar volume of the region defined in Czapla, Swigon, and Olson^14^ by the tolerances *ν*_*ε*_, *τ*_*ε*_ is *ν*_*ε*_*τ*_*ε*_/(2*π*) to leading order, matching the “phase space volume” of 2*ν*_*ε*_*τ*_*ε*_/(4*π*) shown in Czapla, Swigon, and Olson^14^. A comparison to the Shimada-Yamakawa formula in the Supplementary Material serves to further confirm that our normalization matches the standard in the literature.

In our implementation, to reduce the amount of memory required, we archive first-halves and second-halves in files according to their “*z*-bin” value (typically using 128 bins). Once all the half-molecule files are archived, the pairing algorithm works through the second-half files. For each second-half file, a data-structure is set up to sort the molecules into *x*-bins and *y*-bins. At this point, three first-half files are read (those whose *z*-bin match or are one away) one molecule at a time, and for each molecule, its recorded *x*-bins and *y*-bins are used to query the data-structure for all possible matches. At this point, the *z*-difference is compared to *ε* as an easy computation to rule out many pairs as not possibly closing. Only for those pairs that survive these checks is the Euclidean distance between the ends computed and compared to *ε*, and if that test succeeded, then the *SO*(3)-distance is computed and compared to *δ*.

### Fitting functional form to results

For each set of data (*N*_*i*_, *y*_*i*_), where *y*_*i*_ is the value of ${\mathrm{log}}_{10}\,J$ estimated by Monte Carlo for a molecule with *N*_*i*_ basepairs, we perform a least-squares fit to a function of the form

28 $$y=a+b(N-\varphi )+c{[f(N,\varphi ,p)]}^{2},$$

where

29 $$f(N,\varphi ,p)=\,{\rm{mod}}(N-\varphi +\frac{p}{2},p)-\frac{p}{2}.$$

We use the modulo operation “mod” to generate periodic behavior. By definition, mod (*a*, *p*) gives the remainder when the number *a* is divided by *p*. Thus, in the formula above, whenever *N* increases by *p*, the mod result, and hence the value of *f* is unchanged. Putting the expression $N-\varphi +\frac{p}{2}$ inside the mod and subtracting *p*/2 outside the mod ensures that *f* ranges from −*p*/2 to *p*/2 and equals zero when *N* = *ϕ* + *kp* for any integer *k*.

### Data availability

The datasets generated during and/or analysed during the current study are available from the corresponding author on reasonable request.

## Electronic supplementary material

Supplementary Material
