# Supplementary material for: Quantifying the impact of simple DNA parameters on the cyclization J-factor for single-basepair-addition families
Source: Sci Rep. 2018 Mar 20;8:4882. doi: 10.1038/s41598-018-22502-7 (PMC5861124; doi:10.1038/s41598-018-22502-7)
Supplement: Supplementary file 1 — Supplementary Material [file 41598_2018_22502_MOESM1_ESM.pdf]

Supplementary Material for "Quantifying the impact of simple DNA parameters on the cyclization J-factor for single-basepair-addition families" by Yunjin Tong and Robert S. Manning

1 Integration in $SO(3)$

We parametrize $SO(3)$ by a standard "quaternion" approach: each unit vector $\mathbf{q} \in \mathbb{R}^4$ corresponds to the following element of $SO(3)$:

$$\mathbf{R}(\mathbf{q}) = \frac{1}{w^2+x^2+y^2+z^2} \begin{bmatrix} w^2+x^2-y^2-z^2 & 2xy-2wz & 2wy+2xz \\ 2xy+2wz & w^2-x^2+y^2-z^2 & 2yz-2wx \\ 2xz-2wy & 2wx+2yz & w^2-x^2-y^2+z^2 \end{bmatrix}, \tag{1}$$

with $w = q_4$, $x = q_1$, $y = q_2$, and $z = q_3$. This mapping from S^3 (the unit sphere in $\mathbb{R}^4$) to $SO(3)$ is onto but two-to-one, in the sense that $\mathbf{q}$ and $-\mathbf{q}$ describe the same rotation matrix. We also have the important property that if we take the uniform surface measure on S^3, then the mapping (**??**) generates the Haar measure on $SO(3)$ [**?**, Ch. 5].

In order to have a parametrization that lives on an open set rather than a surface, we take any $\vec{c} = (c_1, c_2, c_3)$ in the open unit ball in $\mathbb{R}^3$ and map it to $f(\vec{c}) = (c_1, c_2, c_3, \sqrt{1-(c_1)^2-(c_2)^2-(c_3)^2})$. This defines a one-to-one and onto map from the open unit ball in $\mathbb{R}^3$ to the upper half of S^3 (minus the equator), and therefore to a large portion of $SO(3)$ "centered" at the identity matrix I, namely all of $SO(3)$ minus the image of the equator.

We will thus define a region in $SO(3)$ as the image of some region in $\vec{c}$-space, i.e., as a subset of the unit ball in $\mathbb{R}^3$. Since we want to use Haar measure on $SO(3)$ to measure volumes in $SO(3)$, and since we know that uniform surface measure on S^3 corresponds this Haar measure on $SO(3)$, we have merely to determine what measure in $\vec{c}$-space corresponds to the uniform measure on S^3. For that we compute the Gram determinant of $\{\frac{\partial f}{\partial c_1}, \frac{\partial f}{\partial c_2}, \frac{\partial f}{\partial c_3}\}$. We compute these vectors:

$$\frac{\partial f}{\partial c_1} = \begin{bmatrix} 1 \\ 0 \\ 0 \\ -c_1/\sqrt{1-\|\vec{c}\|^2} \end{bmatrix}, \quad \frac{\partial f}{\partial c_2} = \begin{bmatrix} 0 \\ 1 \\ 0 \\ -c_2/\sqrt{1-\|\vec{c}\|^2} \end{bmatrix}, \quad \frac{\partial f}{\partial c_3} = \begin{bmatrix} 0 \\ 0 \\ 1 \\ -c_3/\sqrt{1-\|\vec{c}\|^2} \end{bmatrix},$$

and therefore, the matrix of the dot products of all pairs of these vectors is:

$$\begin{bmatrix} 1+(c_1)^2/(1-\|\vec{c}\|^2) & c_1c_2/(1-\|\vec{c}\|^2) & c_1c_3/(1-\|\vec{c}\|^2) \\ c_1c_2/(1-\|\vec{c}\|^2) & 1+(c_2)^2/(1-\|\vec{c}\|^2) & c_2c_3/(1-\|\vec{c}\|^2) \\ c_1c_3/(1-\|\vec{c}\|^2) & c_2c_3/(1-\|\vec{c}\|^2) & 1+(c_2)^2/(1-\|\vec{c}\|^2) \end{bmatrix}.$$

The Gram determinant (i.e., the determinant of this 3×3 matrix) simplifies to $1/(1-\|\vec{c}\|^2)$, and thus a uniform integration measure in $\vec{c}$-space uses the square root of this Gram determinant as its probability measure:

$$\text{Volume of a region } R \text{ in } \vec{c}\text{-space} = \int_R \frac{1}{\sqrt{1-\|\vec{c}\|^2}} d\vec{c}.$$

If we want the uniform integration measure that gives total integral 1, then we compute the above integral for R equal to the entire unit ball. This is readily achieved using spherical coordinates:

$$\int_{\text{unit ball in } \mathbb{R}^3} \frac{1}{\sqrt{1-\|\vec{c}\|^2}} d\vec{c} = \int_0^{2\pi} \int_0^{\pi} \int_0^1 \frac{1}{\sqrt{1-\rho^2}} \rho^2 \sin\theta d\rho d\theta d\phi = (2\pi)(2)(\pi/4) = \pi^2.$$

Thus, in order to compute volumes using the Haar measure in $SO(3)$, normalized such that the total volume of $SO(3)$ is one, given a region $S \subset SO(3)$, one can determine the region R in $\vec{c}$-space that maps to S via the composition of f with R and then compute:

$$\text{Volume of} S \subset SO(3) = \int_R \frac{1}{\pi^2\sqrt{1-\|\vec{c}\|^2}} d\vec{c}.$$

This integration theory generates a natural definition of a "neighborhood" of the identity matrix $I \in SO(3)$. Since $\vec{c} = 0$ corresponds to I, for any $\delta > 0$, we take the set of $\vec{c}$ with $\|\vec{c}\| < \delta$; the image of these $\vec{c}$ in $SO(3)$ is a natural notion of the "ball of radius δ about I in $SO(3)$. Given our integration theory, the volume of this ball is

$$\begin{aligned}
\int_{\text{ball of radius } \delta} \frac{1}{\pi^2\sqrt{1-\|\vec{c}\|^2}} d\vec{c} &= \int_0^{2\pi} \int_0^{\pi} \int_0^{\delta} \frac{1}{\pi^2\sqrt{1-\rho^2}} \rho^2 \sin\theta d\rho d\theta d\phi \\
&= \frac{1}{\pi^2}(2\pi)(2)\left[\frac{1}{2}\left(\arcsin\delta - \delta\sqrt{1-\delta^2}\right)\right] \\
&= \frac{2}{\pi}\left(\arcsin\delta - \delta\sqrt{1-\delta^2}\right)
\end{aligned}$$

References

[1] S.M. Lavalle. *Planning Algorithms.* Cambridge University Press, New York, 2006.

2 Supplementary Figures

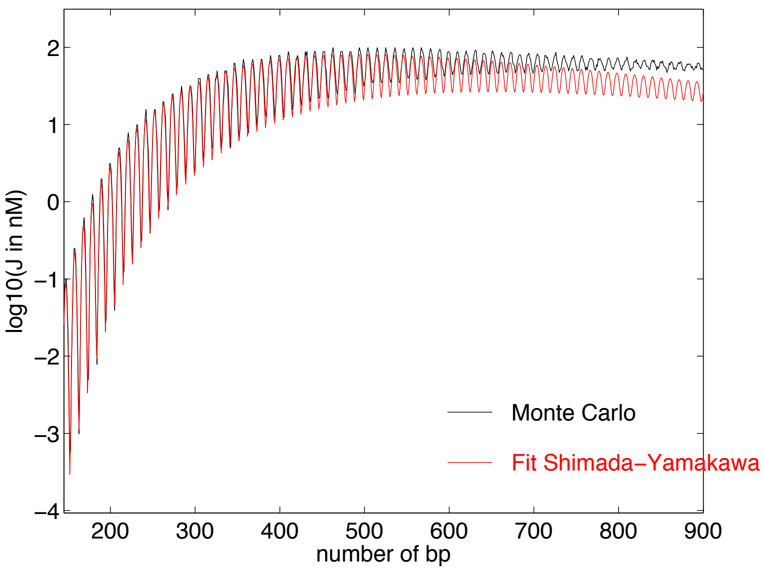

Figure 1: Superposition of MC results with Shimada-Yamakawa for best-fit value of basepair spacing (best fit value is 0.303 nm)

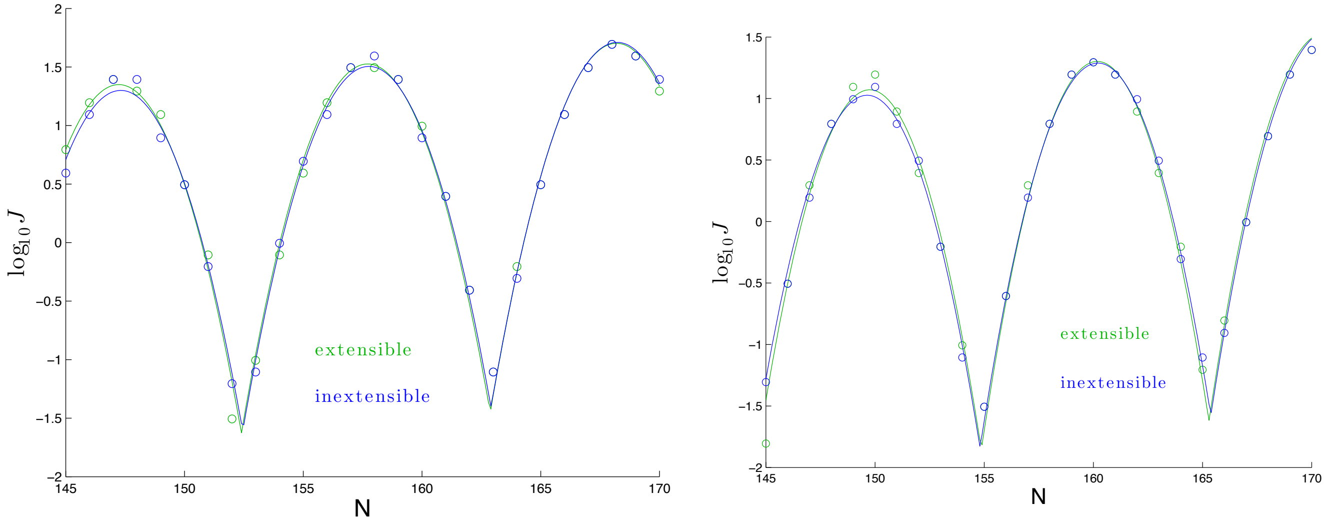

Figure 2: Cyclization profiles for inextensible and extensible models. (Left panel) molecule consists of a planar bend of 90° over 63 bp followed by a straight segment of $N - 63$ bp; (right panel) same construct, but planar bend replaced by a helical bend of 90° with pitch-to-circumference ratio of 1.

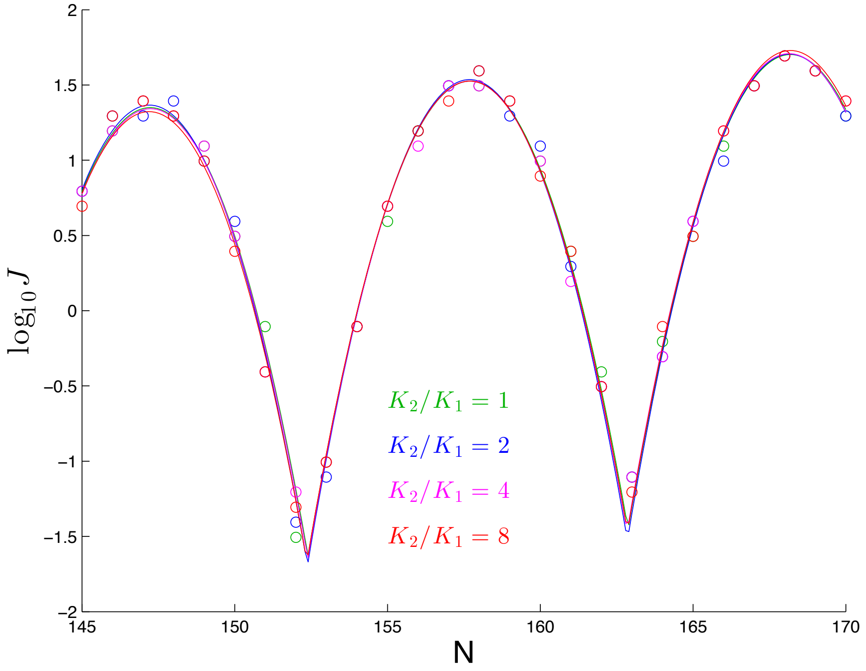

Figure 3: Cyclization profiles as K_2/K_1 varies with the harmonic average $\left[\frac{(K_1)^{-1}+(K_2)^{-1}}{2}\right]^{-1}$ held fixed. Molecules consist of a planar bend of 90° over 63 bp followed by a straight segment of $N - 63$ bp. For $K_2/K_1 = 1$ case, the shared value is $K_2 = K_1 = (46.3/0.34)RT$, consistent with $\beta = 1$ in the main article. For other ratios, the individual values of K_1 and K_2 can be determined from the condition that the harmonic average is maintained at this value $(46.3/0.34)RT$.

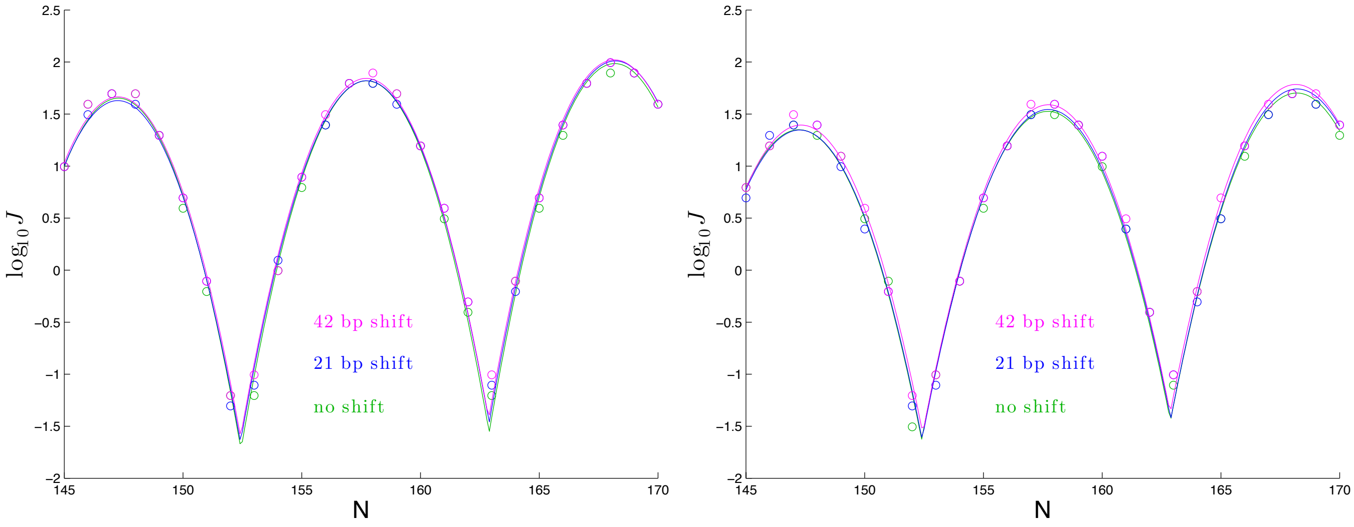

Figure 4: Cyclization profiles as a bend (or pair of bends) is shifted from the start of the molecule to inside the molecule. In the first panel, molecules consist of a k basepair straight segment (for $k = 0, 21, 42$) followed by a a planar bend of 90° over 63 bp followed by a straight segment of $N - 63 - k$ bp. In the right figure, molecules consist of a k basepair straight segment (for $k = 0, 21, 42$), then a 42-bp planar bend of 60°, then a 21-bp straight segment, then a 21-bp planar bend of 30°, then a straight segment of $N - 84 - k$ bp.

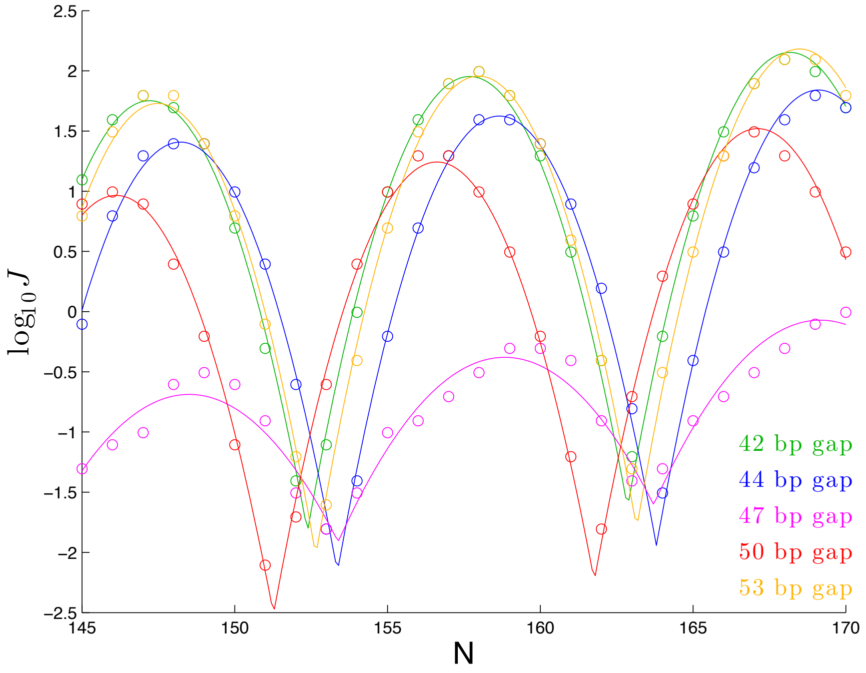

Figure 5: Cyclization profiles as the gap between two bends is varied so as to make them come in and out of phase. Molecules consist of a planar bend of 60° over 42 bp followed by a straight segment of varying lengths followed by a planar bend of 30° over 21 bp, followed by a straight segment. .
